# Evaluating the Effectiveness of Proton Beam Therapy Compared to Conventional Radiotherapy in Non-Metastatic Rectal Cancer: A Systematic Review of Clinical Outcomes

**DOI:** 10.3390/medicina60091426

**Published:** 2024-08-31

**Authors:** Kelvin Le, James Norton Marchant, Khang Duy Ricky Le

**Affiliations:** 1 Melbourne Medical School, The University of Melbourne, Melbourne, VIC 3052, Australia; 2 Department of General Surgical Specialties, The Royal Melbourne Hospital, Melbourne, VIC 3052, Australia; 3 Department of Surgical Oncology, Peter MacCallum Cancer Centre, Melbourne, VIC 3000, Australia; 4 Geelong Clinical School, Deakin University, Geelong, VIC 3220, Australia; 5 Department of Medical Education, Melbourne Medical School, The University of Melbourne, Melbourne, VIC 3000, Australia

**Keywords:** rectal cancer, proton beam therapy, conventional radiotherapy, three-dimensional conformational radiation therapy, volumetric modulated arc therapy, intensity-modulated radiotherapy, toxicity, recurrence, survival

## Abstract

*Background and Objectives*: Conventional radiotherapies used in the current management of rectal cancer commonly cause iatrogenic radiotoxicity. Proton beam therapy has emerged as an alternative to conventional radiotherapy with the aim of improving tumour control and reducing off-set radiation exposure to surrounding tissue. However, the real-world treatment and oncological outcomes associated with the use of proton beam therapy in rectal cancer remain poorly characterised. This systematic review seeks to evaluate the radiation dosages and safety of proton beam therapy compared to conventional radiotherapy in patients with non-metastatic rectal cancer. *Materials and Methods*: A computer-assisted search was performed on the Medline, Embase and Cochrane Central databases. Studies that evaluated the adverse effects and oncological outcomes of proton beam therapy and conventional radiotherapy in adult patients with non-metastatic rectal cancer were included. *Results*: Eight studies were included in this review. There was insufficient evidence to determine the adverse treatment outcomes of proton beam therapy versus conventional radiotherapy. No current studies assessed radiotoxicities nor oncological outcomes. Pooled dosimetric comparisons between proton beam therapy and various conventional radiotherapies were associated with reduced radiation exposure to the pelvis, bowel and bladder. *Conclusions*: This systematic review demonstrates a significant paucity of evidence in the current literature surrounding adverse effects and oncological outcomes related to proton beam therapy compared to conventional radiotherapy for non-metastatic rectal cancer. Pooled analyses of dosimetric studies highlight greater predicted radiation-sparing effects with proton beam therapy in this setting. This evidence, however, is based on evidence at a moderate risk of bias and clinical heterogeneity. Overall, more robust, prospective clinical trials are required.

## 1. Introduction

Rectal cancer is a significant cause of cancer morbidity and mortality [1,2]. For patients with non-metastatic rectal cancer, the current standard of care involves surgery with or without neoadjuvant or adjuvant chemotherapy or radiotherapy [3]. The decision for radiotherapy is determined by tumour features, including the distance from the anal verge, histology and imaging. Management in this way has significantly improved survival and lowered rates of recurrence [4,5,6,7,8,9,10]. Currently, conventional photon-based methods such as three-dimensional conformational radiation therapy (3DCRT) or intensity-modulated radiotherapy (IMRT) are the gold-standard options for rectal cancer radiotherapy. However, data from a radiotherapy trial for rectal cancer between the years 1994 and 2002 suggest that over 70% of patients receiving these therapies report adverse effects, with approximately 25% of those reporting grade 3+ radiotherapy adverse events including diarrhoea and dermatologic and haematologic complications due to off-target irradiation [11,12]. These can lead to significant outcomes for patients, including bowel and bladder dysfunctions, sexual dysfunction, infertility, bone marrow failure, immunosuppression and poorer quality of life [11,13,14,15]. Newer-generation photon-based therapy devices have improved beam concentrating ability, and with improved guidelines dictating threshold radiation doses, there has been significant improvements in rates of radiotoxicity. Currently, it is estimated that grade 3+ adverse events in patients with local rectal cancer occur at a rate between 12.6% and 26.5% [16]. Despite these outcomes, radiotoxicity continues to significantly impact patients with rectal cancer, with over 10% of patients being hospitalised for complications secondary to radiotherapy [17]. 

Proton beam therapy (PBT) has emerged over as a potential alternative to current photon-based radiotherapy. Over 100 operational proton therapy centres exist and are primarily located within the United States of America (USA), and there are plans for the development of an additional 60 facilities worldwide [18,19]. In Australia, the Australian Bragg Centre for Proton Therapy and Research will be the first location within the Southern Hemisphere to offer this therapy. PBT has been touted as a highly appealing radiotherapy option for rectal cancer with the ability to minimise radiation dose to tissue and therefore minimise treatment-related radiotoxicity [18]. This is achieved through principles of the Bragg Peak, which describes the dose deposition of protons within a narrow, well-defined range. This allows for increased irradiation at the cancer site whilst simultaneously limiting exposure to surrounding organs [20,21,22,23,24]. In theory, the reduction in offset irradiation exposure garners potential to minimise both acute and late toxicity to surrounding organs and reduce long-term treatment-related sequelae [25]. Superior treatment outcomes and reduced toxicity have been observed in clinical trials using PBT versus conventional radiotherapy for several cancers involving those of the gastrointestinal tract, head and neck region and for those in paediatric populations [24,26,27,28]. The current efficacy and safety profile of PBT compared to current radiotherapy remains poorly characterised due to the relative novelty of PBT and the infancy of the infrastructure surrounding its access. This systematic review aims to explore the adverse effects, assess dosimetric comparisons and evaluate the oncological outcomes associated with PBT versus conventional radiotherapy for non-metastatic rectal cancer.

## 2. Materials and Methods

### 2.1. Review Protocol and Registration

This review was performed in adherence to the Preferred Reporting Items for Systematic Reviews and Meta-Analyses (PRISMA) guidelines [29]. The review protocol was prospectively registered in the PROSPERO database (CRD42024543201).

### 2.2. Literature Search

A literature search of the Medline, EMBASE and Cochrane CENTRAL databases was performed on 2 May 2024. The reference lists of relevant articles were used to identify additional articles. The search strategy combined keywords and medical subject headings (MeSH) terms related to proton beam therapy, radiotherapy (standard photon beam) and rectal cancer. Subject terms, truncations and Boolean operators were used during this process to find all relevant articles. The search query is available in Appendix A.

### 2.3. Inclusion and Exclusion Criteria

Full-text, peer-reviewed comparative studies available in the English language which evaluated adults with non-metastatic rectal cancer treated with PBT or conventional radiotherapy were considered for inclusion in this review. The Union for International Cancer Control (UICC) TNM classification (8th edition) of malignant tumours was used to define cancer stages [30].

Publications were included if they were original and peer-reviewed randomised controlled trials or prospective or retrospective cohort studies, included adults (age > 18) with non-metastatic (non-stage IV) rectal cancer identified prior to commencement of radiotherapy, compared PBT to conventional photon beam radiotherapy and evaluated outcomes of interest. 

Publications were excluded if they were of the following study types: reviews, meta-analyses, conference papers or posters, non-human trials, letters, opinion articles, abstracts, commentaries, case reports and case series. Articles were also excluded if they explored paediatric (age < 18 years) populations, had incomplete data, evaluated metastatic rectal cancer (stage IV) or other cancers including those of the colon and anus and utilised other modalities of radiotherapy such as but not limited to carbon ion therapy.

### 2.4. Literature Screening

Initial screening by title and abstract was performed by two independent investigators (KL and JNM) for consideration of progression to full-text analysis. Articles where there was insufficient information also proceeded to full-text analysis. Following title and abstract screening, the same two investigators (KL and JNM) independently conducted full-text analysis for consideration of inclusion. Disagreement during this process was resolved by discussion with consensus. If consensus was not achieved, a third independent investigator (KDRL) was consulted for final decision. 

### 2.5. Outcomes

The primary endpoints of this review were related to adverse treatment or radiotoxicity outcomes, including dysuria, rectal bleeding, lymphopenia, proctitis, diarrhoea, pain, cystitis, fistula, bowel obstruction, discontinuation of treatment, bone marrow failure and radiation dose. Secondary outcomes that were explored included oncological outcomes such as recurrence, overall survival, cancer-specific survival and progression-free survival.

### 2.6. Data Extraction

Eligible studies were extracted for data including author, year, study design, country of publication, demographic data of included cohorts (age, sex and number of patients), data related to rectal cancer (tumour stage and overall grade) as well as data related to outcomes of interest. 

### 2.7. Quality and Risk of Bias Assessment

Assessments of methodological rigour and quality of included studies were performed by two independent investigators (KL and JNM) using the Risk of Bias in Non-randomised Studies of Interventions (ROBINS-I) tool [31]. Disagreement during this process was resolved with discussion in consensus. If consensus was not achieved, a third independent investigator (KDRL) was consulted for final decision.

### 2.8. Data Synthesis

Statistical analysis was conducted utilising Review Manager 5.4 (RevMan 5.4) (Cochrane, London, UK) software. Odds ratios (OR) and their 95% confidence intervals (95% CI) were extracted from the included studies or calculated where required. To undertake meta-analysis, homogeneous data were pooled where possible. In cases of no heterogeneity, a fixed-effects model was used to determine size of effect. If there was heterogeneity, a random-effects model was used. If there were heterogeneous reports of continuous data, where relevant, they were converted to single measures of effect, such as from median and interquartile range to mean and standard error, utilising the Wan method [32]. For meta-analysis, pooling of patient outcomes was performed for each outcome of interest and reported with Forest plots where relevant. For simple statistical comparisons between continuous variables, a two-tailed paired Student’s *t*-test was performed where relevant. Heterogeneity of included studies was evaluated with the Higgins I^2^ test, where values <25% were considered of low heterogeneity, those between 25 and 50% were considered as moderate and those >50% were considered as high heterogeneity [33]. A *p*-value of <0.05 was considered to be statistically significant. In the absence of homogeneous data, descriptive results of relevant outcomes were reported.

### 2.9. Subgroup Analysis

Subgroup analyses and/or meta-regression were performed to investigate sources of heterogeneity where relevant. In this study, planned subgroup analyses included analysis of outcomes following removal of papers deemed at serious or critical risk of bias and analysis of outcomes for lower-grade (T1-2) and higher-grade (T3-4) tumours. 

## 3. Results

### 3.1. The Results of the Literature Search 

**Figure 1:**
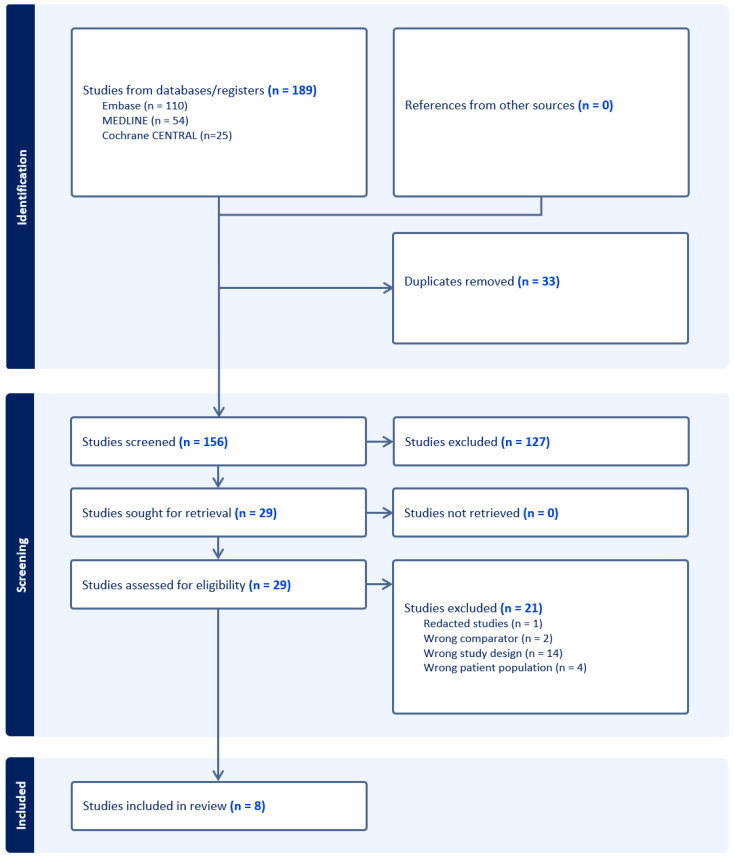
Search strategy and workflow in accordance with PRISMA guidelines.

A total of 189 articles were retrieved following a computer-assisted search. Following the removal of duplicates, 156 articles underwent title and abstract screening by two independent investigators (KL and JNM) to assess for eligibility, resulting in the exclusion of 127 articles. A full-text analysis was subsequently performed on the remaining 29 articles based on our eligibility criteria. A total of 21 articles were excluded in this process, which included 14 publications with the wrong study designs, 2 studies testing comparators not of interest, 4 studies exploring the wrong patient population and 1 retracted study. The PRISMA flow chart is shown in Figure 1.

### 3.2. Overview of Included Studies

A total of eight studies were included in this study (Table 1) [34,35,36,37,38,39,40,41]. All eight studies assessed dosimetric comparisons between PBT and differing types of conventional radiotherapy, including 3DCRT, IMRT and volumetric modulated arc therapy (VMAT). One study used dosimetric comparisons, albeit with microtron accelerators, to generate models of tumour control (tumour control probability; TCP) and tissue complication (normal tissue complication probability; NTCP) [35]. One study conducted a single-arm, non-randomised clinical trial exploring adverse effects including recurrence, metastases and survival rates with PBT [37]. The studies were predominately conducted within Europe (n = 6). Cohort sizes were variable, ranging from n = 6 to n = 25. Tumour grading across all studies ranged from T2-T4. Tumour staging ranged from stages IIa-III.

### 3.3. Adverse Treatment Outcomes

There was insufficient data to allow for a pooled comparative analysis of the primary outcomes of interest related to adverse treatment outcomes between PBT and conventional radiotherapy treatment. However, one study involving 15 patients explored acute and late toxicities from PBT in a single-arm non-randomised trial [37]. Toxicity grading was retrospective, and severity was determined using the Common Terminology Criteria for Adverse Events (CTCAE) version 4.0 scale [37]. Additionally, eight studies involving 105 patients explored and compared radiation exposure in surrounding organs with PBT and conventional radiotherapy dosimetry plans [34,35,36,37,38,39,40,41]. Of these, five studies involving 70 patients were available for a pooled analysis for the following subgroups: pelvis, sacrum, bowel and bladder [34,37,39,40,41]. The parameters used for radiation exposure were either the mean dose exposure (Gy), max dose exposure (Gy), VXGy or the volume of the organ receiving at least X% of the intended prescribed dose (%), and DX% or the dose received by X% of the volume (Gy).

#### 3.3.1. Acute Toxicity

Moningi et al. reported that 66.7% of patients with rectal cancer (n = 10/15) that underwent PBT developed grade 1–2 acute toxicities [37]. Grade 1 toxicities included fatigue (n = 8/15; 53.3%), diarrhoea (n = 6/15; 40%), pain (n = 5/15; 33.3%), nausea (n = 4/15; 26.7%), constipation (n = 2/15, 13.3%), dermatitis (n = 2/15, 13.3%), headache (n = 1/15; 6.7%) and hematochezia (n = 1/15; 6.7%). Grade 2 toxicities included pain (n = 2/15, 13.3%), dermatitis (n = 1/15; 6.7%), diarrhoea (n = 1/15; 6.7%) and mucositis (n = 1/15; 6.7%). The authors also reported one incident (n = 1/15; 6.7%) of grade 3 lymphopenia. Aside from this, there were no other cases of grade 3+ acute toxicities observed. All patients (n = 15) were able to complete the prescribed PBT treatment without requiring treatment breaks, and there were no episodes of treatment discontinuation [37]. No studies compared PBT and conventional radiotherapy for rates of dysuria, proctitis, cystitis, fistula, bowel obstruction and bone marrow failure. 

#### 3.3.2. Late Toxicity

Moningi et al. reported three incidences (n = 3/15; 20%) of late toxicity, defined as adverse events occurring six or more weeks after PBT [37]. Late toxicities included grade 1 pain and grade 3 dysuria in one patient (n = 1/15, 6.7%), grade 3 rectal bleeding in another (n = 1/15, 6.7%) and a grade 2 pelvic fracture in one patient (n = 1/15, 6.7%) [37].

#### 3.3.3. Radiation Exposure from Dosimetric Analyses

##### Pelvis

Two studies involving 23 patients were pooled for an analysis of radiation exposure at the pelvis in a random-effects model [34,37]. This demonstrated lower predicted radiation exposure at the pelvis for PBT compared to 3DCRT at the V5Gy (MD = −28.94%; 95% CI = −50.18 to −7.7; *p* = 0.008), V10Gy (MD = −28.34%; 95% CI = −32.66 to −24.02; *p* < 0.00001) and V20Gy (MD = −21.71%; 95% CI = −43.08 to −0.34; *p* = 0.05) dose levels [34,37]. Although unable to be pooled, Colaco et al. 2014 also highlighted reduced predicted exposure from PBT compared to IMRT when measuring V5Gy to V20Gy (n = 8, all comparisons at *p* = 0.0156) [34]. Pedone et al. further demonstrated strong evidence of reduced predicted radiation exposure to the pelvis when comparing PBT to VMAT at the V5Gy to V15Gy parameters (all comparisons at *p* < 0.001) [38]. 

##### Sacrum

Two studies involving 22 patients were pooled for an analysis of radiation exposure at the sacrum in a random-effects model [39,40]. This analysis demonstrated no difference in the mean predicted dose exposure between PBT and VMAT plans (MD = −1.4 Gy; 95% CI = −2.87 to 5.68; *p* = 0.52) [39,40]. When interpreted individually, Radu et al. highlighted higher predicted mean exposures (*p* = 0.0004) and dose radiation at D50% (*p* = 0.0002) in PBT compared to VMAT plans (n = 7) [39]. Additionally, Kronborg et al. demonstrated that PBT was associated with a lower median radiation dose to the sacrum and sacroiliac joints at V20Gy compared to IMRT with a median of 46.70 compared to 76.60, respectively [36]. 

##### Bowel

Three studies involving 47 patients were pooled for an analysis of radiation exposure at the bowel in a random-effects model [39,40,41]. This analysis showed very strong evidence of a reduced mean dose exposure with PBT compared to VMAT plans (MD = −13.5 Gy; 95% CI = −18.7 to −8.3; *p* < 0.00001) [39,40,41]. Additionally, two studies involving 32 patients also had sufficient data for a subgroup analysis to be performed. This further highlighted lower expected dose exposures in the bowel in PBT compared to VMAT at the D50% (MD = −20.01 Gy; 95% CI = −25.83 to −14.20; *p* < 0.00001) and D1% (MD = −1.11 Gy; 95% CI = −1.83 to −0.39; *p* = 0.003) parameters [39,41]. Three articles additionally explored the dose-sparing potential of PBT. Colaco et al. observed reduced expected exposure to the small bowel in PBT compared to 3DCRT when measuring V10Gy to V40Gy (n = 8, with all comparisons at *p* = 0.0156), and in PBT compared to IMRT only from V10Gy to V20Gy (n = 8, with all comparisons at *p* = 0.0156) [34]. Moningi et al. demonstrated decreases in the predicted exposure to the bowel in PBT compared to 3DCRT plans at the V30Gy parameter (n = 15; *p* = 0.0017) [37]. Pedone et al. found lower predicted exposures in PBT plans compared to VMAT at the bowel bag at doses from V5Gy to V15Gy (n = 20, *p* < 0.001), but not at V25Gy [38]. 

##### Bladder

Three studies involving 47 patients were pooled for an analysis of the predicted radiation exposure at the bladder in a random-effects model [39,40,41]. This strongly demonstrated a decreased mean radiation exposure in PBT compared to VMAT plans (MD = −10.95 Gy; 95% CI = −18.49 to −3.42; *p* = 0.004) [39,40,41]. Two studies involving 32 patients were additionally analysed. This analysis showed very strong evidence to suggest a reduction in the expected exposure to the bladder in PBT plans compared to VMAT at the D50% parameter (MD = −16.69 Gy; 95% CI = −21.43 to −11.95; *p* < 0.00001), but not at the D1% parameter (MD = 3.16 Gy; 95% CI = −3.86 to 10.19; *p* = 0.38). Three articles with non-homogeneous data further evaluated the dose-sparing potential of PBT. Colaco et al. showed strong evidence of a lower predicted reduction in dose exposure in PBT plans compared to 3DCRT when measuring at V40Gy (n = 8, *p* = 0.0016), but not at V50Gy [34]. Pedone et al. found strong evidence of lower predicted exposures in PBT plans compared to VMAT plans at the bladder when measured from V5Gy to V15Gy (n = 20, with all comparisons at *p* < 0.001), but not at V25Gy [38]. Moningi et al. showed no significant differences in the expected exposure between PBT and 3DCRT [37].

##### Other Structures

Three studies explored the dose-sparing effects of PBT on the femoral head. Rønde et al. found strong evidence of a reduced expected dose exposure with PBT versus VMAT in both the ipsilateral and contralateral femoral head (n = 15, *p* < 0.001) [40]. Moningi et al. similarly demonstrated a lower predicted max dose exposure in PBT compared to 3DCRT plans (n = 7; left femur, *p* = 0.0018; right femur, *p* = 0.0018) [37]. Finally, Radu et al. showed very strong evidence of lower mean dose exposures (left femur, *p* < 0.0001; right femur, *p* = 0.0001) and at the D50% parameter (left femur, *p* < 0.0001; right femur, *p* = 0.0003) in PBT plans compared to VMAT (n = 7) [39]. 

Two studies investigated the predicted radiation exposure on several genitourinary structures. When comparing PBT and VMAT, Rønde et al. demonstrated lower predicted mean dose exposures at the contralateral ureter (*p* < 0.001) and penile bulb (*p* = 0.008); however, there were no differences at the ipsilateral ureter and vagina (n = 15) [40]. Wolff et al. showed lower predicted exposures at the testes in terms of the mean dose and at D1% and D50% between PBT and RapidArc, IMRT and 3DCRT (n = 25, with all comparisons at *p* < 0.05) [41].

One study analysed dose exposure at the anus. Wolff et al. found no significant differences in the dose exposure in PBT plans compared to RapidArc, IMRT and 3DCRT [41]. 

One study explored the predicted radiation exposure at the L5-S2 nerve root. Radu et al. found very strong evidence of lower mean dose exposures (*p* < 0.0001) and at the D50% parameter (*p* = 0.0002) at the L5-S2 nerve roots in PBT versus VMAT plans (n = 7) [39]. 

##### Models for Tumour Control

One study involving six patients used dosimetric comparisons to generate models of TCP and NTCP [35]. PBT exhibited higher TCP values across various clonogenic cell densities, with 75% at *p* = 10^7^, 64% at *p* = 10^8^ and 51% at *p* = 10^9^ compared to 64%, 51%, and 38% for X-ray therapy at the same respective densities. Mixed-therapy plans demonstrated intermediate TCP values: 71% at *p* = 10^7^, 59% at *p* = 10^8^ and 46% at *p* = 10^9^ [35]. In terms of NTCP, both PBT and mixed-therapy plans allowed for higher doses (63–92 Gy and 61–87 Gy, respectively) compared to X-ray therapy (60–80 Gy). A comparative NTCP analysis showed that PBT resulted in a 7.5% reduction in the risk of normal tissue damage compared to X-ray therapy when optimised to a 5% complication probability in any risk organ. The small bowel was the primary organ at risk for 83.3% of patients (n = 5/6). Proton therapy and mixed plans provided better sparing of the small bowel compared to X-rays, with bladder risk being at 1% for proton therapy, 0% for X-ray therapy and 4.67% for mixed-therapy plans [35]. Importantly, these results from Isaacson et al. were derived from therapy beams delivered by a microtron, the latter which has been superseded by more advanced particle accelerators.

### 3.4. Oncological Outcomes

There are currently no prospective, randomised-controlled trials that compare the oncological outcomes of PBT and conventional radiotherapy treatment. One study explored long-term oncological outcomes with PBT in a single-arm non-randomised trial (n = 15) [37]. No studies have explored cancer-specific survival.

#### 3.4.1. Local and Distant Recurrence

Moningi et al. reported both local and distant recurrence following PBT re-irradiation. Specifically, 20% of patients (n = 3/15) developed local recurrence only, 13.3% (n = 2/15) developed distant metastases only and 13.3% of patients (n = 2/15) developed both local recurrence and distant metastases [37]. All patients who developed distant metastases (n = 4; 26.7%) died. Among those with local recurrence, one patient (n = 1; 6.7%) died of unknown cause. Another patient (n = 1; 6.7%) without local recurrence died of aggressive acute leukaemia 9 months after completing PBR re-irradiation. The median follow-up time after PBT irradiation was 13.9 months [37].

#### 3.4.2. Overall Survival

Moningi et al. reported a median overall survival from the start of re-irradiation of 39.0 months (range 0.5–38.9), with both the 1-year and 2-year overall survival rates being 67.5% (n = 10) [37]. 

#### 3.4.3. Progression-Free Survival

Moningi et al. reported that the median progression-free survival was 15.0 months (range 0.5–36.2). The 1-year progression-free survival rate was 58.7% (n = 9), and the 2-year progression-free survival rate was 47% (n = 7) [37]. 

### 3.5. Results from Quality and Risk of Bias Assessment

**Figure 2:**
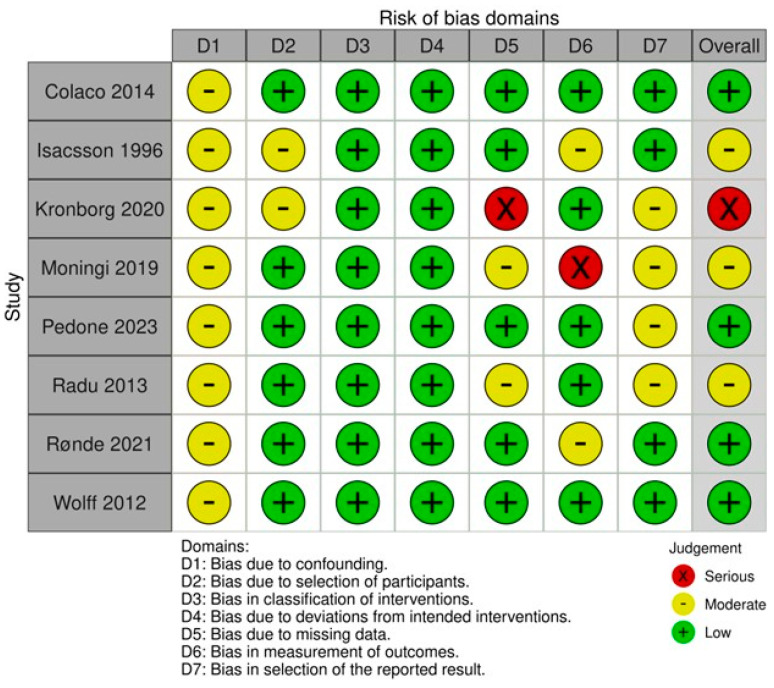
Quality assessment of included articles utilising the ROBINS-I assessment tool [34,35,36,37,38,39,40,41].

Overall, four articles (50%) were considered to have a low risk of bias, three articles (37.5%) were considered to have a moderate risk of bias and one article (12.5%) was considered to have a serious risk of bias (Figure 2). The key areas that were considered to introduce bias included domains of confounding factors, selection bias and bias secondary to missing data. All articles (n = 8) were assessed to have potential confounding factors due to having a small sample size ranging from 6 to 25 patients, which limits statistical power and generalisability. Two articles were deemed to have moderate risks of bias from patient selection due to a lack in explicit justification for cohort inclusion and unjustified, unequal distribution of PBT and conventional radiotherapy groups. Three articles were deemed to have a moderate-to-severe risk of bias due to missing data, including data on the tumour grade and stage, age and sex distribution. The lack of a statistical analysis, limitations from a single-arm trial and isolated reporting of specific subgroups of the total included patient population were factors deemed to introduce moderate-to-severe outcome measurement bias to three articles and bias from the reported results in four articles.

## 4. Discussion

This systematic review revealed insufficient evidence to suggest that PBT is superior to conventional radiotherapy for reducing adverse treatment and radiotoxicity outcomes or improving oncological outcomes for patients with non-metastatic rectal cancer. On the contrary, there has been extensive research to suggest PBT is associated with reduced off-set radiation exposure through dosimetric analyses. We demonstrate that PBT reduces the radiation dose at the pelvis, bowel, bladder and other structures including the femoral head, several genitourinary structures and surrounding nerve bodies. This was not the case for the sacrum. These results align with current evidence ascertaining the dose-sparing effects of PBT compared to conventional radiotherapies in comparative dosimetric plans [18]. 

Importantly, our results occur on the background of studies at an overall moderate risk of bias. Furthermore, the included studies have significant clinical heterogeneity. In particular, despite our pooled analyses showing reduced radiation dosages with PBT compared to conventional radiotherapy plans, comparisons were made between PBT and VMAT for the bowel, bladder, femoral head and sacrum, but comparisons were made between PBT and 3DCRT for the femoral head and pelvis. Furthermore, within these regions, the results also varied depending on the radiation dose plan. Additionally, upon the examination of individual studies, further limitations must also be noted. For example, although Kronborg et al. reported reduced sacral irradiation with PBT plans, their dosimetric analysis was conducted in the context of determining ideal radiation doses to minimise pelvic insufficiency fracture and therefore differs in aim and approach compared to the other dosimetric comparisons within this review [36]. Furthermore, Isaccson et al. utilised therapies delivered through a microtron, a technology that has since been superseded and is no longer used in clinical radiation oncology. Overall, the heterogeneity in these cross-modality comparisons therefore introduces an element of bias into the overall interpretation of the reported findings. Therefore, the results of this review should be considered with caution. 

The current management for very early to intermediate non-metastatic rectal cancers involves oncological resection with or without neoadjuvant or adjuvant treatment radiotherapy and/or chemotherapy [30,42]. This paradigm in treatment has led to significant improvements in survival and reduced rates of recurrence [3]. PBT has garnered interest as an alternative to conventional radiotherapy to minimise radiation-induced toxicity due to its unique Bragg peak, which defines the narrow dose deposition curve at the tumour site. This enhances the precision of radiation to the tumour and, in turn, minimises the dose exposure to surrounding tissues and organs at risk [12,22]. Several dosimetric studies analysing non-metastatic and metastatic rectal cancers similarly document dose reduction at surrounding organs at risk in PBT plans compared to conventional radiotherapy [43,44]. Extending from rectal cancers, PBT has similarly shown dose-sparing effects to surrounding tissues in several other malignancies involving the gastrointestinal system, prostate, head and neck, central nervous system as well as paediatric cancers [21,22,23,24]. This systematic review confirms the dose-sparing effects of PBT compared to conventional radiotherapies. However, it remains a challenge to ascertain the potential benefits of PBT in reducing adverse effects due to the paucity in current evidence [45]. 

Conventional radiotherapy remains challenged with radiotoxicities [12,46]. Several studies showed rates of higher-grade toxicities in 25% of patients, which can lead to debilitating physical problems and impair social functioning [11,47,48]. Overall, PBT was documented to primarily result in low-grade acute toxicities with few incidences of grade 3+ acute toxicity and late toxicity events [37]. Similar outcomes were reported in four studies involving patients with non-metastatic and metastatic rectal cancer, with two studies reporting no cases of grade 2+ toxicity following PBT treatment [43,44,49,50]. This mild toxicity profile is potentially distinct from conventional radiotherapy but must be further explored in robust comparative studies to determine any beneficial effects. In terms of oncological outcomes, there remains the same challenges with the paucity of robust data. The current clinical data on recurrence outcomes in non-metastatic and metastatic rectal cancers such as rates of subsequent metastasis after treatment are variable [37,44,50]. This may be attributed to the significant clinical heterogeneity between studies, evidenced by the many confounding factors including cancer grade and stage, follow-up times and other interpatient variabilities, which are inconsistently reported amongst different studies. In addition, measures of overall survival approximate around 70%, although the data are quite variable and are based on small sample sizes and therefore may not be generalisable [37,49,50]. One study found similar rates of overall survival (n = 55; 65%) following preoperative radiotherapy, which proves comparable to the current reported data [51]. Another consideration that remains unexplored according to the current evidence are outcomes related to PBT irradiation for recurrent rectal cancer. Recent evidence suggests that PBT irradiation is associated with poor 2-year overall survival (60%), progression-free survival (10%) and local control (58.3%) [52]. Furthermore, the same study identified the rate of grade 1 adverse events to be 70% and that of grade 3+ events to be 10% [52]. Similarly, this evidence is based on a small sample size (n = 10). Overall, there is a need for direct, appropriately powered comparative studies to further characterise the outcomes of PBT compared to conventional radiotherapy. 

In the clinical setting, PBT has been offered for many cancers, especially for the management of central nervous system, head and neck, prostate and paediatric malignancies across Europe and the USA [22,53,54]. Several studies have highlighted superior treatment outcomes and reduction in toxicity for other cancers, such as oesophageal malignancy, head and neck cancers, low-grade gliomas and paediatric chordoma and chondrosarcoma [24,26,27,28,55]. However, the main limitations of PBT include cost and accessibility. PBT is currently still considered an emerging technology, with over 100 proton centres around the world and approximately 60 being planned for implementation, and less than 1% of patients who undergo expected radiotherapy treatments undergo PBT [18]. Additionally, the costs of PBT facility implementation are notably high. The cost of developing three to four PBT treatment room centres can exceed USD 100–200 million, whereas single-room facilities can cost up to USD 30 million [18]. These costs are magnitudes greater compared to those of conventional radiotherapy centres [18]. Moreover, due to the scarcity of PBT facilities, accessibility proves a challenge. For example, in a study conducted in the USA, Amini et al. found that most patients in a pool of over 100,000 patients had travelled over 100 miles to a treatment facility [54]. Given these barriers, there are strict regulatory guidelines that dictate access to PBT as well as tight criteria for access to PBT via clinical trials. For jurisdictions challenged by limitations to healthcare spending, or for certain populations such as those from rural and remote areas, the practicality of PBT remains an area of ongoing consideration, and potential inequity exists. Finally, the patient costs for PBT treatment are notably higher upfront, especially when accounting for long-distance travelling and potential overseas travel [18,56]. However, through minimising toxicity and improving oncological outcomes, many offer the opinion that the net costs associated with PBT may prove lower when accounting for transport, accommodation, the management of toxicity, allied health services and other opportunity costs associated with conventional radiotherapy [56]. 

This systematic review provides a further update to the field in ascertaining the potential benefits of PBT in rectal cancer. However, there are some limitations to note. Firstly, a priori adverse effects and oncological outcomes could not be systematically reviewed due to the paucity of evidence comparing PBT and conventional therapy for patients with non-metastatic rectal cancer. In the literature, only one study reported adverse effects (acute and late toxicity) and oncological outcomes in a single-arm non-randomised trial involving PBT. However, this study involved a small sample size (n = 15) and was deemed to have a moderate risk of bias; therefore, the reported results are not generalisable or comparable with conventional radiotherapy and should be interpreted with caution (Figure 2). Due to this, comparative analyses and conclusions regarding the safety and efficacy of PBT in comparison to conventional radiotherapy could not be made. The only adverse effect in which a pooled analysis was conducted was radiation exposure. However, it is important to acknowledge that all studies (n = 8) had low sample sizes with a max of n = 25, an overall moderate risk of bias as well as marked clinical heterogeneity. Furthermore, there was inconsistent reporting of demographic information amongst the included studies. This limited control for confounding demographic factors in the analysis of outcomes within this study and did not allow for the analysis of potentially important subgroups. Of these, the analysis of outcomes based on covariates such as tumour grade and stage was particularly not possible. These subgroups may significantly impact outcomes related to radiotherapy treatment and, therefore, decisions as to which radiotherapy is most appropriate [18]. Additionally, there remains a paucity in the current literature. This was evident after our search in the Medline and Embase databases such that we progressed to a search in Cochrane CENTRAL, thus representing an a priori deviation. Furthermore, there is a noticeable lack of comparative studies between 1996 and 2012. It is likely that this inactivity is due to time needed for the development of infrastructure in light of technological advancement, including the advent of pencil beam scanning beam delivery, passive scatter techniques and more advanced planning systems [36,37]. Furthermore, there is likely a time lag bias in the reporting of outcomes that is also attributed to the time needed to recruit patients and analyse data. Overall, these limitations underscore the necessity for more comprehensive, direct comparative studies to provide a clearer understanding of the toxicity profiles, benefits and risks associated with PBT. Whilst the dosimetric analysis showed strong evidence of harm minimisation with low statistical heterogeneity, these data may not translate clinically to improvements in quality of life, longer-term oncological outcomes or reductions in toxicity in clinical scenarios [45]. For example, there have been instances where PBT showed no significant benefit over conventional radiation, such as in paediatric malignancies, and it has even led to severe toxicities such as in brain malignancies despite several studies documenting the benefits of PBT. This may be due to reasons including patient and/or tumour heterogeneity or a complex anatomy, which are potential significant factors in deciding on the type of radiotherapy [18]. It is likely that as PBT becomes increasingly adopted and more accessible, results from current and future comparative trials will become available to better characterise the efficacy and safety outcomes.

## 5. Conclusions

This systematic review demonstrated insufficient evidence to prove the superiority of PBT compared to conventional radiotherapy in minimising adverse treatment outcomes and improving oncological outcomes for patients with non-metastatic rectal cancer due to the paucity of current data. However, a pooled dosimetric analysis demonstrated reductions in the radiation dose exposure to surrounding tissue with PBT. These findings, however, are formed on the basis of few studies with high clinical heterogeneity and an overall moderate risk of bias. Therefore, the clinical translatability may not necessarily reflect the superiority of PBT over conventional radiotherapy. There is a need for more robust, prospective, randomised clinical trials with larger sample sizes to inform the guidelines related to PBT use in non-metastatic rectal cancer. 

## Figures and Tables

**Table 1:** Overview of included studies.

Author	Year	Country of Publication	Study Design	Total Patients (n)	Age (Median; Range)	Sex (M:F)	Tumour Grade	Tumour Stage (UICC-TMN)	Treatment
Colaco et al. [34]	2014	USA	Comparative dosimetric study	8	NR	NR	T2-T3	NR	PBT, 3DCRT, IMRT
Isacsson et al. [35]	1996	Sweden	Comparative dosimetric study	6	60 (47–79)	4:2	T4	III	PBT, X-ray, Mixed
Kronborg et al. [36]	2020	Denmark	Comparative dosimetric study	9	69 (35–81)	5:4	T3-T4	IIa-III	PBT, 3DCRT, IMRT, VMAT
Moningi et al. [37]	2019	USA	Comparative dosimetric study and retrospective single-arm non-randomised trial	15	74 (55–91)	8:7	NR	NR	PBT, 3DCRTPBT reirridiation
Pedone et al. [38]	2023	Sweden	Comparative dosimetric study	20	57 (36–73)	12:8	T2-T4	IIb-III	PBT, VMAT
Radu et al. [39]	2013	Sweden	Comparative dosimetric study	7	64 (52–75)	6:1	T4	III	PBT, VMAT
Rønde et al. [40]	2021	Denmark, Norway	Comparative dosimetric study	15	NR	8:7	NR	NR	PBT, 3DCRT,
Wolff et al. [41]	2012	Germany	Retrospective comparative dosimetric study	25	62.5 (44–73)	15:10	T2-T4	IIa-III	PBT, 3DCRT, IMRT, RapidArc

## Data Availability

No new data were generated or developed in the work related to this manuscript. All referenced data are publicly available on medical databases as described in our Methodology Section.

## Appendix A. Search Query

1. exp Rectal Neoplasms/
2. ((rectal or rectum or recti or pararect* or anal or anus or perianal) adj5 (adenoma* or cancer* or carcinoma* or tumo* or malign* or neoplas* or mass)).tw,kf.
3. 1 or 2
4. Proton Therapy/
5. exp Radiotherapy, Computer-Assisted/
6. PBT.tw,kf.
7. XRT.tw,kf.
8. photon*.tw,kf.
9. proton*.tw,kf.
10. 4 or 6 or 9
11. 5 or 7 or 8
12. 3 and 10 and 11
